# Assessing network degeneration and phenotypic heterogeneity in genetic frontotemporal lobar degeneration by decoding FDG-PET

**DOI:** 10.1016/j.nicl.2023.103559

**Published:** 2023-12-22

**Authors:** Nick Corriveau-Lecavalier, Leland R. Barnard, Scott A. Przybelski, Venkatsampath Gogineni, Hugo Botha, Jonathan Graff-Radford, Vijay K. Ramanan, Leah K. Forsberg, Julie A. Fields, Mary M. Machulda, Rosa Rademakers, Ralitza H. Gavrilova, Maria I. Lapid, Bradley F. Boeve, David S. Knopman, Val J. Lowe, Ronald C. Petersen, Clifford R. Jack, Kejal Kantarci, David T. Jones

**Affiliations:** a Department of Neurology, Mayo Clinic Rochester, USA; b Department of Psychiatry and Psychology, Mayo Clinic Rochester, USA; c Department of Quantitative Health Sciences, Mayo Clinic Rochester, USA; d Department of Neuroscience, Mayo Clinic Jacksonville, USA; e VIB-UA Center for Molecular Neurology, VIB, University of Antwerp, Belgium; f Department of Medical Genetics, Mayo Clinic Rochester, USA; g Department of Radiology, Mayo Clinic Rochester, USA

**Keywords:** Clinical neurology, Frontotemporal dementia, Frontotemporal lobar degeneration, FDG-PET, Machine learning

## Abstract

Genetic mutations causative of frontotemporal lobar degeneration (FTLD) are highly predictive of a specific proteinopathy, but there exists substantial inter-individual variability in their patterns of network degeneration and clinical manifestations. We collected clinical and ^18^Fluorodeoxyglucose-positron emission tomography (FDG-PET) data from 39 patients with genetic FTLD, including 11 carrying the *C9orf72* hexanucleotide expansion, 16 carrying a *MAPT* mutation and 12 carrying a *GRN* mutation. We performed a spectral covariance decomposition analysis between FDG-PET images to yield unbiased latent patterns reflective of whole brain patterns of metabolism (“eigenbrains” or EBs). We then conducted linear discriminant analyses (LDAs) to perform EB-based predictions of genetic mutation and predominant clinical phenotype (i.e., behavior/personality, language, asymptomatic). Five EBs were significant and explained 58.52 % of the covariance between FDG-PET images. EBs indicative of hypometabolism in left frontotemporal and temporo-parietal areas distinguished *GRN* mutation carriers from other genetic mutations and were associated with predominant language phenotypes. EBs indicative of hypometabolism in prefrontal and temporopolar areas with a right hemispheric predominance were mostly associated with predominant behavioral phenotypes and distinguished *MAPT* mutation carriers from other genetic mutations. The LDAs yielded accuracies of 79.5 % and 76.9 % in predicting genetic status and predominant clinical phenotype, respectively. A small number of EBs explained a high proportion of covariance in patterns of network degeneration across FTLD-related genetic mutations. These EBs contained biological information relevant to the variability in the pathophysiological and clinical aspects of genetic FTLD, and for offering valuable guidance in complex clinical decision-making, such as decisions related to genetic testing.

## Highlights

• Large-scale network degeneration is heterogeneous across FTLD-related genetic mutations.
• This variability can be linearly parametrized by a small set of latent factors ("eigeinbrains") derived from FDG-PET.
• These eigenbrains contain relevant information associated with FTLD-related pathogenic mechanisms and clinical syndromes.
• This supports the utility of data-driven techniques to develop tools supporting clinical decision making in genetic FTLD.

## 1 Introduction

Frontotemporal lobar degeneration (FTLD) is one of the most common causes of dementia in individuals of age 65 and younger (Rabinovici and Miller, 2010, Knopman and Roberts, 2011, Boeve et al., 2022). A notable feature of this class of degenerative diseases is the relatively high proportion of cases for which the culprit of clinical symptoms is purely genetic, which is estimated to be 10 % to 30 % of all patients with frontotemporal dementia (FTD) (Goldman et al., 2005, Goldman et al., 2008, Rohrer et al., 2009, Moore et al., 2020, Ramos et al., 2020). The three major genes implicated in genetic FTLD are microtubule-associated protein tau (*MAPT*), progranulin (*GRN*) and chromosome 9 open reading frame 72 (*C9orf72*), where *MAPT* mutations are associated with a primary tauopathy and *GRN* and *C9orf72* are associated with TDP-43 pathology (Panza et al., 2020). A clinico-pathological paradox in genetic FTLD is that genetic mutations are highly predictive of a specific underlying proteinopathy, however they can all emerge into a wide variety of clinical syndromes predominantly targeting behavioral and personality and/or language, as well as memory and/or motor functions, although to a lower frequency (Bang et al., 2015, Moore et al., 2020, Boeve et al., 2022). This syndromic diversity is tied to specific patterns of large-scale network degeneration, which are equally heterogeneous across FTLD-related genetic mutations (Peet et al., 2021, Staffaroni et al., 2022).

An increasing number of studies provide empirical support for the use of data-driven techniques to unravel the clinico-radiological heterogeneity of degenerative dementia syndromes. For instance, a recent study by (Jones et al., 2022) using a spectral covariance decomposition of ^18^Fluorodeoxyglucose positron emission tomography (FDG-PET) images to show that a latent space representation indexes patterns of network degeneration associated with a wide range of dementia syndromes. This approach has also been utilized to decipher the clinico-radiological heterogeneity of relatively circumscribed dementia syndromes such as posterior cortical atrophy (Townley et al., 2021) and dysexecutive Alzheimer’s disease (Corriveau-Lecavalier et al., 2023b). In FTLD, similar methods have been used to assess patterns of network degeneration associated with the heterogeneity of clinical symptoms in behavioral variant fronto-temporal dementia (bvFTD) (Ranasinghe et al., 2021, Ramanan et al., 2023) and semantic dementia (Ramanan et al., 2023) and to delineate transcriptomics vulnerability and connectomics factors associated with atrophy patterns in sporadic and genetic bvFTD (Shafiei et al., 2023). Taken together, these findings highlight the potential of data-driven methodologies to extricate the complex relationships between underlying proteinopathy, patterns of network degeneration and syndromic diversity in genetic FTLD. The potential use of these techniques could provide valuable information to identify patterns of network degeneration indicative of specific genetic mutations, track disease progression and assess risk of phenoconversion, and develop novel tools informed by quantitative neuroimaging to guide complex clinical decision making (Barnard et al., 2022, Vogel et al., 2022).

In the present investigation, we aimed to assess patterns of network degeneration in genetic FTLD using machine learning. To this end, we applied a spectral decomposition of covariance between FDG-PET images in a cohort of individuals with a confirmed genetic mutation causative of FTLD (i.e., *MAPT*, *GRN*, *C9orf72*). This analysis produced latent patterns of network degeneration based on glucose metabolism that are unbiased by clinical classification or genetic status. We then evaluated how these patterns distinguished between FTLD-related genetic mutations and associated clinical syndromes using group-wise comparisons and linear discriminants analyses (LDAs).

## 2 Material and methods

### 2.1 Participants

Mutation carriers were recruited from Mayo Clinic Rochester clinical practice and were co-enrolled in the Alzheimer’s Disease Research Center (ADRC) and ARTFL LEFFTDS Longitudinal Frontotemporal Lobar Degeneration (ALLFTD) programs. All of them had a confirmed genetic mutation causative of FTLD (*MAPT*, *GRN*, *C9orf72*) (see procedure below). All clinical diagnoses were rendered by neurologists subspecialized in behavioral neurology using clinical standards adopted by experts in the field. The diagnostic process primarily relied on medical history obtained from the patient and a reliable informant and neurological examination including cognitive screening using the Short Test of Mental Status (Kokmen et al., 1991). Assessments were conducted through a structured interview covering various cognitive and behavioral symptoms related to the core clinical criteria for the clinical syndromes included in the study. Additional diagnostic assessment, including imaging, neuropsychological and/or speech and language pathology assessments, were often conducted as part of clinical care or co-enrollment in research programs. While these assessments supported clinical diagnoses, they did not determine it. Because neuropsychological and speech and language assessments were heterogeneous (e.g., performed in outside settings versus at Mayo Clinic, clinical versus research settings, etc.) and not administered across the whole patient cohort, we do not include such data in the paper. Carriers either had normal neurologic function (i.e., asymptomatic) or a clinical diagnosis of an FTLD-related syndrome predominantly and initially targeting behavior and/or language according to widely accepted criteria (Gorno-Tempini et al., 2011, Rascovsky et al., 2011). Although the statistical analyses only considered the predominant clinical phenotype, a more fine-grained clinical classification of these phenotypes includes bvFTD (Rascovsky et al., 2011), mild behavioral and/or cognitive impairment (MCBI) (Barker et al., 2022), both of which may be accompanied with parkinsonism (P), motor-neuron disease (MND) or amyotrophic lateral sclerosis (ALS), and primary progressive aphasia (PPA) (Gorno-Tempini et al., 2011) and language mild cognitive impairment (langMCI), both of which could be accompanied by corticobasal syndrome (CBS) (Armstrong et al., 2013). A description of the diagnostic process and fine-grained phenotypic information about symptomatic carriers can be found in Supplementary Table 1.

This study met HIPAA privacy standards and was approved by the Mayo Clinic Institutional Review Board. Patients and/or their legal representative provided written consent upon their clinical visit for their data to be used for research purposes.

### 2.2 Genetic testing

Study participants were screened for mutations related to genetic FTLD (*C9orf72*, *GRN*, *MAPT*) based on DNA extracted from peripheral blood according to previously established protocols (Hutton et al., 1998, Baker et al., 2006, DeJesus-Hernandez et al., 2011, Ramos et al., 2020).

### 2.3 FDG-PET acquisition and processing

FDG-PET images were acquired using a PET/CT scanner (GE healthcare or Siemens) after a 30-minute uptake period in a dimly lit room. Scanning acquisition lasted 8 min divided into four 2-minute dynamic frames following a low-dose CT transmission scan. Imaging processing was done using an MRI-free pipeline including the registration to the Mayo Clinic Adult Lifespan Template (MCALT) (available at https://www.nitrc.org/projects/mcalt/) using a non-linear symmetric diffeomorphic registration, a spatial normalization using a 6 mm full-width-half-minimum (FWHM) kernel and intensity normalization to the pons to produce a standard uptake value ratio (SUVR) image.

### 2.4 Biological projection and reduction

We performed a spectral decomposition of covariance between FDG-PET images called “Biological projection and reduction” (BPR), which provides a biologically interpretable, low-dimensional state space reflecting inter-individual variability in patterns of macro-scale network degeneration (Townley et al., 2021, Jones et al., 2022, Corriveau-Lecavalier et al., 2023b). This method consists of mean-centering FDG-PET images and scaling them by the interquartile range (IQR), and flattening images into a one-dimension array of voxels which are then entered into a subject-by-voxel matrix. This matrix is then submitted to a singular value decomposition to yield a set of latent factors, or “eigenbrains” (EBs). It is important to note that EBs do not represent patterns of hypometabolism per se, but rather patterns of whole-brain metabolism with opposing poles of relative hyper- and hypometabolism. The directionality of patient-level patterns is determined by the loading factor on a given eigenbrain, referred as to an “eigenvalue”. This eigenvalue can be either positive or negative and describes how the pattern of hypometabolism in a given patient relates to the topology and directionality of an EB. The number of EBs to retain for analysis was determined using Horn’s method (Horn, 1965). This method stems from the sampling theory and proposes that factoring should stop when components account for equivalent or lower variance than expected by chance. This is determined using the latent-root criterion, which compares the latent root of each EB to those of randomly determined variables with identical dimensions.

### 2.5 Statistical analysis

Analyses were performed with a combination of *Python* (version 3.7.12) (BPR analysis) and *R* (version 4.0.5; https://www.rproject.org/) (statistical analyses). Demographic and clinical data between symptomatic carriers grouped according to genetic mutation (*MAPT*, *GRN*, *C9orf72*) and asymptomatic carriers (regardless of mutation) were compared using analyses of variance (ANOVAs) followed by Tukey’s post-hoc tests when the omnibus test was significant (continuous variables) and chi square analyses (categorical variables).

Differences between genetic mutations and clinical syndromes (i.e., behavioral, language, asymptomatic) on each significant EB were assessed using ANOVAs. Mixed phenotypes (e.g., mixed bvFTD/PPA) were classified according to the initial clinical manifestation. We performed a multivariable linear model to assess the relationship between significant EBs and cognitive impairment. Specifically, all eigenvalues from significant EBs were entered as predictors of STMS scores across the entire cohort. Finally, we conducted linear discriminant analyses (LDAs) based on eigenvalues of all significant EBs to perform data-driven multiclass predictions separately for the type of genetic mutation and clinical phenotype. These predictions were then compared with true genetic mutation and clinical syndromes.

## 3 Results

### 3.1 Demographic and clinical data

Table 1 displays demographic and clinical data for the patient sample. Age FDG-PET differed across groups, where all symptomatic carriers groups were older than asymptomatic carriers, and *C9orf72* and *GRN* mutation carriers were older than *MAPT* mutation carriers. Groups also differed at age at symptom onset, where *GRN* carriers were older than both *C9orf72* and *MAPT* carriers, and *C9orf72* carriers were older than *MAPT* carriers. Asymptomatic carriers had higher STMS scores than *GRN* carriers only. However, groups did not statistically differ in terms of sex distribution or years of education. The most frequent predominant phenotype across mutations was behavioral (25/39). Most carriers with a predominant language phenotype had a *GRN* mutation (6/7) and most asymptomatic carriers had a *MAPT* mutation (6/7). Due to the very small number of persons with specific mutations in *MAPT* and *GRN*, information on specific mutations is purposefully not included here in order to protect confidentiality.

**Table 1:** Demographic and clinical data.

	*C9orf72*	*MAPT*	*GRN*	Asymptomatic	*P* value
*N*	11	10	11	7	
Age at FDG	59.6 (10.6)	50.3 (11.9)	66.2 (5.33)	41.4 (6.3)	<0.001^a^
Age at symptom onset	52.5 (12.4)	42.2 (8.08)	63.1 (6.09)	N/A	<0.001^b^
Sex (M, F)	9, 2	6, 4	8, 3	3, 4	0.3
Education (years)	15.6 (1.99)	14.2 (1.92)	17.4 (2.16)	16 (2.38)	0.08
STMS (/38)	27.7 (9.85)	26.5 (6.95)	24.4 (11.6)	37 (0.58)	0.02^c^

a Asymptomatic < *C9orf72*, *GRN*, *MAPT; MAPT < GRN*, *MAPT.*

b *GRN* > *C9orf72 > MAPT*.

c *GRN* < Asymptomatic. STMS = Short Test of Mental Status.

### 3.2 Eigenbrains results

**Fig. 1:**
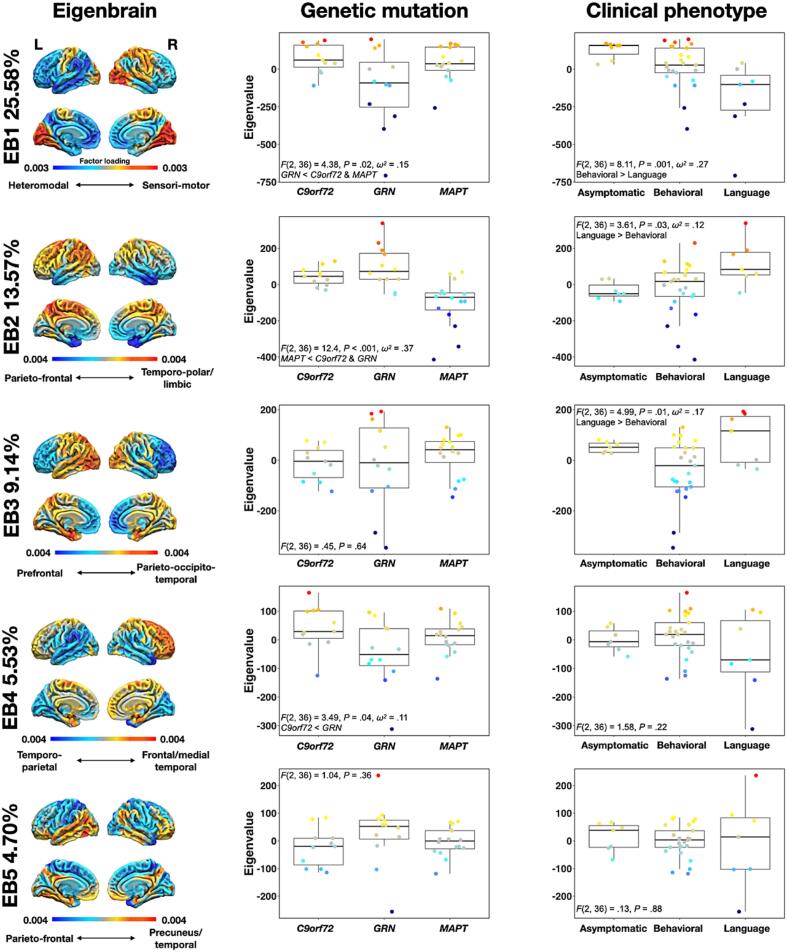
FDG-PET eigenbrains and group comparisons according to genetic mutation and predominant clinical phenotype. The color bars represent positive (warm colors) and negative (cold colors) eigenvalues for each EB. These EBs reflect patterns of relative metabolism between two opposing poles of hyper- and hypometabolism. For each EBs, a negative eigenvalue is associated with lower metabolism in areas of cold colors relative to those with warm colors. EB = eigenbrain. EB = Eigenbrain.

Five EBs were retained for analysis and collectively accounted for 58.52 % of covariance between FDG-PET images. A visual depiction of the Horn analysis used to determine the optimal number of EBs can be found in Supplementary Fig. 1. Fig. 1 displays these EBs along with group-wise comparisons between genetic mutations and predominant clinical phenotype. Supplementary Fig. 1 displays the same data along with fine-grained phenotypic information.

EB1 accounted for 25.58 % of covariance and reflected a pattern of cortical organization opposing relative metabolism in heteromodal cortices bilaterally with a right hemispheric predominance (negative eigenvalues) to primary sensory and motor areas bilaterally (positive loading). Group-wise comparisons according to the type of genetic mutation revealed that *GRN* had lower eigenvalues compared to *MAPT* and *C9orf72* mutations carriers. Comparisons according to predominant clinical phenotype showed that carriers with language-associated syndromes had lower eigenvalues compared to those with predominant behavioral syndromes and asymptomatic carriers. This means that more hypometabolism in heteromodal cortices relative to primary and sensorimotor areas was associated with a higher likelihood of having a *GRN* mutation and a language-related phenotype relative to their genetic and phenotypic counterparts.

EB2 accounted for 13.57 % of covariance and reflected a pattern opposing relative metabolism in medial frontal, orbitofrontal, and temporopolar areas bilaterally with a strong right hemispheric predominance (negative eigenvalues) to parieto-frontal areas bilaterally with a left hemispheric dominance (positive eigenvalues). Group-wise comparisons according to the type of genetic mutation revealed that *MAPT* had lower eigenvalues compared to *GRN* and *C9orf72* mutations carriers. This means that *MAPT* mutation carriers had more hypometabolism in medial/orbitofrontal and temporopolar areas relative to parieto-frontal cortices compared to *GRN* and *C9orf72* mutations carriers, and vice-versa. Group-wise comparisons according to predominant clinical phenotype showed that carriers with language-associated syndromes had higher eigenvalues compared to those with predominant behavioral syndromes and asymptomatic carriers, and thus had more hypometabolism in parieto-frontal cortices relative to medial/orbitofrontal and temporopolar areas relative to other phenotypes.

EB3 accounted for 9.14 % of covariance and reflected a pattern opposing relative metabolism in prefrontal areas bilaterally with a right hemispheric predominance and right middle and inferior temporal cortices (negative eigenvalues) to occipito-parietal areas bilaterally with a slight left hemispheric dominance and left temporal cortices (positive eigenvalues). Group-wise comparisons between clinical phenotypes revealed that carriers with language-related disorders had higher eigenvalues than those with a predominant behavioral syndrome. This means that carriers with language-related disorders had relatively more hypometabolic patterns in left occipito-parieto-temporal areas compared to right prefrontal areas compared to behavioral phenotypes, and vice-versa. Comparisons between genetic mutations were not significant.

EB4 accounted for 5.53 % of covariance and reflected a pattern opposing relative metabolism in lateral temporo-parietal areas with a right hemispheric predominance (negative eigenvalues) to frontal and medial temporo-parietal areas (positive eigenvalues). Group-wise comparisons according to the type of genetic mutation revealed that *C9orf72* had lower eigenvalues compared to *GRN* mutation carriers. Thus, *C9orf72* mutation carriers had, on average, more hypometabolism in right lateral temporo-parietal areas relative to frontal and medial temporo-parietal areas compared to *GRN* mutations carriers, and vice-versa. Group-wise comparisons according to predominant clinical phenotype were not significant.

Eigenbrain 5 (EB5) accounted for 4.70 % of covariance and reflected patchy patterns opposing relative metabolism in parieto-frontal areas bilaterally (negative eigenvalues) to temporal areas with a left hemispheric predominance, the precuneus, and the orbitofrontal cortex bilaterally and the right dorsolateral prefrontal cortex (positive eigenvalues). Group-wise comparisons according to the type of genetic mutation or predominant clinical phenotypes were not significant.

### 3.3 Associations between EBs and cognitive impairment

A multivariable linear model including eigenvalues of all significant EBs as predictors of STMS scores revealed that lower eigenvalues on EB1 (i.e., hypometabolism in heteromodal cortices relative to primary and sensorimotor areas) predicted lower STMS scores, i.e., more severe cognitive impairment, *F(5, 30) = 4.03, p = 0.0065, R^2adj^ = 0.302*.

### 3.4 LDA multi-class predictions

**Fig. 2:**
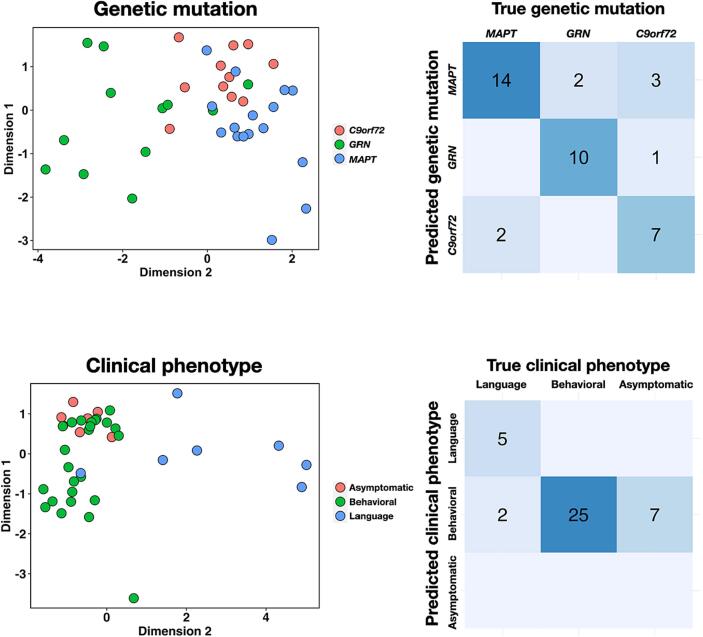
Linear discriminant analysis embedding based on FDG-PET eigenbrains. Carriers are represented in the embeddings based on the LDA analyses separately conducted for genetic mutation and predominant clinical phenotype. Confusion matrices are shown comparing true versus predicted category for genetic mutation and predominant clinical phenotypes.

The LDA analyses aimed at predicting genetic mutation or predominant clinical phenotype used two linear discriminants in both cases. Results are displayed in Table 2. The two-dimensional embeddings resulting from these LDA analyses along with confusion matrices between true and predicted labels are displayed on Fig. 2.

**Table 2:** Performance metrics for the linear discriminant analyses for genetic mutation and predominant clinical phenotype.

	Genetic mutation		
Metric	*C9orf72*	*MAPT*	*GRN*
Sensitivity	0.778	0.909	0.737
Specificity	0.867	0.929	0.900
Positive predictive value	0.636	0.833	0.875
Negative predictive value	0.929	0.963	0.783
Balanced accuracy	0.822	0.919	0.818

	Predominant clinical phenotype		
Metric	Behavioral	Language	Asymptomatic

Sensitivity	0.735	1.000	N/A
Specificity	1.000	0.941	0.821
Positive predictive value	1.000	0.714	N/A
Negative predictive value	0.357	1.000	N/A
Balanced accuracy	0.868	0.971	N/A

The multi-class predictions yielded an accuracy of 79.5 % (confidence intervals 63.5–90.7 %) for genetic status. Predictions were mostly accurate for each genetic mutation, where 14/16 *MAPT*, 10/12 *GRN* and 7/11 *C9orf72* carriers were correctly classified. Classification accuracy for predominant clinical phenotype was 76.9 % (confidence intervals 60.7–88.9 %). Here, 5/7 language and all of the behavioral phenotypes were correctly classified, whereas all asymptomatic cases were misclassified as behavioral. The two mislabeled language phenotypes were predicted to be behavioral, and none of the cases were predicted to be asymptomatic.

## 4 Discussion

We aimed to decipher the clinico-radiological heterogeneity of genetic FTLD using data-driven techniques. To this end, we used a spectral decomposition analysis to decode the covariance between FDG-PET images of a cohort of patients with one of the three major genetic mutations that cause FTLD. This yielded five latent patterns of network degeneration, which are referred to as EBs, that are reflective of this heterogeneity and that explained nearly 60 % of the covariance between FDG-PET images across the whole patient cohort. These EBs contained biological information relevant to inter-individual variability in clinical symptomatology and predicted genetic mutation with relatively high accuracy. These findings have important implications for the phenotypic and biological characterization of FTLD-related genetic mutations and the development of clinical decision-making tools to track disease progression and risk of phenoconversion in genetic FTLD.

Our findings using FDG-PET images as a starting point are in large agreement with previous studies which have used group-wise comparisons between clinical syndromes and genetic mutations (Whitwell et al., 2012). An important observation is that EBs reflecting asymmetry in patterns of metabolism (EB1, EB2, EB4) distinguished *GRN* mutation carriers from other genetic mutations. This aligns with the common observation of asymmetric patterns of brain abnormalities in this population, which are often left-lateralized (Rohrer et al., 2010, Chen and Kantarci, 2020, Saracino et al., 2023). Moreover, these left-lateralized hypometabolic patterns, which involved the parieto-frontal areas (i.e., “executive control network”) and the temporo-parietal junction (i.e., “language network”), were most often associated with clinical phenotypes predominantly targeting language functions (Whitwell et al., 2015). This is in line with investigations that have found a greater prevalence of language-related phenotypes in *GRN* mutation carriers relative to other common FTLD genetic mutations (Le Ber et al., 2008, Van Langenhove et al., 2013, Moore et al., 2020, Saracino et al., 2021). The finding of a significant association between eigenvalues on EB1 and STMS scores also echo previous studies from our group showing that the degeneration of heteromodal cortices better relates to higher-order cognitive processes rather than behavioral symptoms (Corriveau-Lecavalier et al., 2022, Jones et al., 2022, Corriveau-Lecavalier et al., 2023b, Corriveau-Lecavalier et al., 2023a).

Hypometabolic patterns in frontotemporal areas with a strong involvement of temporal poles with a right hemispheric predominance (EB2) were highly specific to *MAPT* mutation carriers and were associated with predominantly behavioral phenotypes. This is again in line with previous studies showing that *MAPT* mutations most often target the temporal lobes (Cash et al., 2018, Fumagalli et al., 2018, Jones et al., 2018, Chu et al., 2021, Staffaroni et al., 2022). This also underlines the crucial yet underappreciated involvement of temporopolar areas not only in language functions but also in emotional regulation and behavior (Josephs et al., 2009, Erkoyun et al., 2020, Younes et al., 2022, Mesulam, 2023). This also indicates that clinical phenotypes caused by *MAPT* mutations may resemble those associated with sporadic FTLD selectively targeting temporal poles and which are almost universally caused by TDP-43 type C (Josephs et al., 2009, Mesulam, 2023, Mesulam et al., 2023). This may warrant different therapeutic strategies in individuals manifesting this pattern depending on whether it is caused by a *MAPT* mutation or not.

Another finding is that hypometabolism in frontotemporal areas (EB3) was associated with predominant behavioral phenotypes. This was expected given that this pattern is highly similar to the archetypical pattern of atrophy observed in bvFTD (Whitwell et al., 2011, Boeve, 2022). This pattern was however not specific to any genetic mutation. This is likely due to the fact that bvFTD is the most common phenotype across all genetic mutations that cause FTLD (Moore et al., 2020), and hence this pattern of abnormalities lacks specificity in that regard. Regarding *C9orf72* carriers, relative hypometabolism in frontal and parietal areas (EB2 and EB4) discriminated these carriers from *GRN* and *MAPT* carriers. However, there was no clear correspondence with a predominant clinical phenotype. This may be due to the relatively higher clinical heterogeneity in *C9orf72* included in this group relative to other mutations, and the fact that *C9orf72* carriers more often manifest with prominent psychiatric and motor features (Snowden et al., 2012), which were not considered in this study. Further investigation of data-driven patterns of network degeneration in relation to these symptoms are needed.

The LDA analyses strictly using eigenvalues as input features achieved relatively high accuracy in predicting genetic mutation status. This suggests that FTLD-related genetic mutations, despite their substantial clinico-radiological heterogeneity, have specific patterns of network degeneration that is encoded in their large-scale pathophysiology and that can be detected with FDG-PET. Prediction of predominant clinical phenotype was excellent for symptomatic carriers. This is in line with previous studies showing that FDG can distinguish degenerative dementia phenotypes with high accuracy and can provide valuable information for differential diagnosis (Nestor et al., 2018, Jones et al., 2022). All asymptomatic carriers were misclassified as having a behavioral phenotype, which significantly hindered the overall model performance. This may, however, indicate that FDG allows for detection of early pathognomonic metabolic changes prior to overt clinical symptomatology. It is, however, to be determined whether these carriers will eventually evolve into a behavioral syndrome upon longitudinal follow-up.

Overall, our findings show that data-driven techniques applied to FDG-PET imaging were able to quantify patterns of network degeneration associated with the clinical heterogeneity across major FTLD genetic mutations. Moreover, these patterns largely align with the extant literature that have used group-wise comparisons to compare clinical and radiological features across these mutations. This has wide-ranging clinical implications. A better understanding of the patterns of network degeneration specific to FTLD-related genetic mutations can guide clinical decision-making. For instance, it can hint at the presence of an FTLD-related genetic mutations and prompt further workup including genetic testing, if not already planned. This is particularly important given the relatively high frequency of fully penetrant genetic mutations in FTLD compared to other common causes of degenerative dementia. The integration of such knowledge into tools aimed at supporting complex clinical decision-making informed by the quantification of FDG-PET is underway (Barnard et al., 2022). However, it is important to note that the current study did not include non-genetic FTLD patients and hence the sensitivity and specificity of such patterns of degeneration to FTLD-related genetic mutations remains to be determined. Our findings also support the development of network-based biomarkers utilizing global patterns of network degeneration to potentially track disease progression and risk of phenoconversion across FTLD-related genetic mutations. Such biomarkers, either in isolation or in combination with other tools such as plasma biomarkers, could improve disease progression models and optimize clinical trials, for instance by reducing sample size required to detect clinically meaningful effects induced by therapeutic interventions (Staffaroni et al., 2022). This is an active line of research from our group. It is also important to emphasize the advantages of data-driven techniques that parametrize inter-individual variability in the global physiology of the brain as a starting point rather than a priori clinical classifications or even genetic status in isolation. Indeed, many recent studies have shown that large-scale patterns of global function that are selectively degenerated in dementia syndromes align with many relevant biological properties such as the transcriptomic, myeloarchitectonic, and cytoarchitectonic topology of the brain (Burt et al., 2018, Huntenburg et al., 2018, Shafiei et al., 2023) and neurotransmission systems (Goulas et al., 2021, Hansen et al., 2022). A better understanding of the large-scale patterns of degeneration across FTLD-related genetic mutations could thus help develop effective interventions aimed at large-scale systems.

Our findings must be interpreted in the context of a few limitations. The sample size is rather small, which directly speaks to the low prevalence of FTLD-related genetic mutations. This prevented us from examining patterns of network degeneration associated with a wider range of clinical manifestations. This study is cross-sectional, and therefore it was not possible to assess how patterns of network degeneration change over time. This would be particularly important in asymptomatic carriers to assess whether changes in network physiology can forecast impending phenoconversion. The data included in this study came from both clinical practice and research settings, and hence clinical examinations were not homogeneously conducted. Finally, while our analyses allowed us to uncover about 60 % of the covariance between FDG-PET images, around 40 % remains to be explained. Future studies will be needed to investigate factors potentially related to this unexplained variance (e.g., cognitive reserve, technical factors, etc.).

## 5 Conclusions

We leveraged data-driven methodologies to uncover the clinico-radiological heterogeneity of the three major genetic mutations that cause FTLD. A small set of latent patterns of large-scale network degeneration accounted for a high proportion of covariance between FDG-PET images and related to the syndromic variability across these genetic mutations. These findings are important to better understand the large-scale physiology of genetic FTLD and have implications for the development of disease models, interventions programs, and the development of clinical tools supported by quantitative imaging. Longitudinal multimodal studies in large cohorts covering a wider phenotypic spectrum and asymptomatic carriers are required to fulfill these aims.

## CRediT authorship contribution statement

**Nick Corriveau-Lecavalier:** Conceptualization, Data curation, Formal analysis, Investigation, Methodology, Project administration, Software, Writing – original draft, Writing – review & editing. **Leland R. Barnard:** Conceptualization, Data curation, Formal analysis, Methodology, Software, Writing – review & editing. **Scott A. Przybelski:** Data curation, Methodology, Writing – review & editing. **Venkatsampath Gogineni:** Conceptualization, Data curation, Methodology, Writing – review & editing. **Hugo Botha:** Conceptualization, Methodology, Supervision, Writing – review & editing. **Jonathan Graff-Radford:** Conceptualization, Methodology, Writing – review & editing. **Vijay K. Ramanan:** Investigation, Writing – review & editing. **Leah K. Forsberg:** Data curation, Investigation, Project administration, Writing – review & editing. **Julie A. Fields:** Investigation, Writing – review & editing. **Mary M. Machulda:** Methodology, Writing – review & editing. **Rosa Rademakers:** Investigation, Writing – review & editing. **Ralitza H. Gavrilova:** Investigation, Writing – review & editing. **Maria I. Lapid:** Investigation, Writing – review & editing. **Bradley F. Boeve:** Conceptualization, Funding acquisition, Investigation, Methodology, Project administration, Resources, Supervision, Writing – review & editing. **David S. Knopman:** Conceptualization, Funding acquisition, Investigation, Methodology, Project administration, Resources, Supervision, Visualization, Writing – original draft, Writing – review & editing. **Val J. Lowe:** Investigation, Methodology, Resources, Writing – review & editing. **Ronald C. Petersen:** Funding acquisition, Investigation, Project administration, Resources, Writing – review & editing. **Clifford R. Jack:** Funding acquisition, Investigation, Methodology, Resources, Supervision, Writing – review & editing. **Kejal Kantarci:** Conceptualization, Funding acquisition, Investigation, Methodology, Project administration, Resources, Supervision, Writing – review & editing. **David T. Jones:** Conceptualization, Funding acquisition, Investigation, Methodology, Project administration, Resources, Supervision, Visualization, Writing – original draft, Writing – review & editing.

## Declaration of competing interest

The authors declare that they have no known competing financial interests or personal relationships that could have appeared to influence the work reported in this paper.

## Data Availability

Data will be made available on request.
