# Supplementary material for: Assessing network degeneration and phenotypic heterogeneity in genetic frontotemporal lobar degeneration by decoding FDG-PET
Source: Neuroimage Clin. 2023 Dec 22;41:103559. doi: 10.1016/j.nicl.2023.103559 (PMC10944211; doi:10.1016/j.nicl.2023.103559)
Supplement: Supplementary data 1 [file mmc1.docx]

**
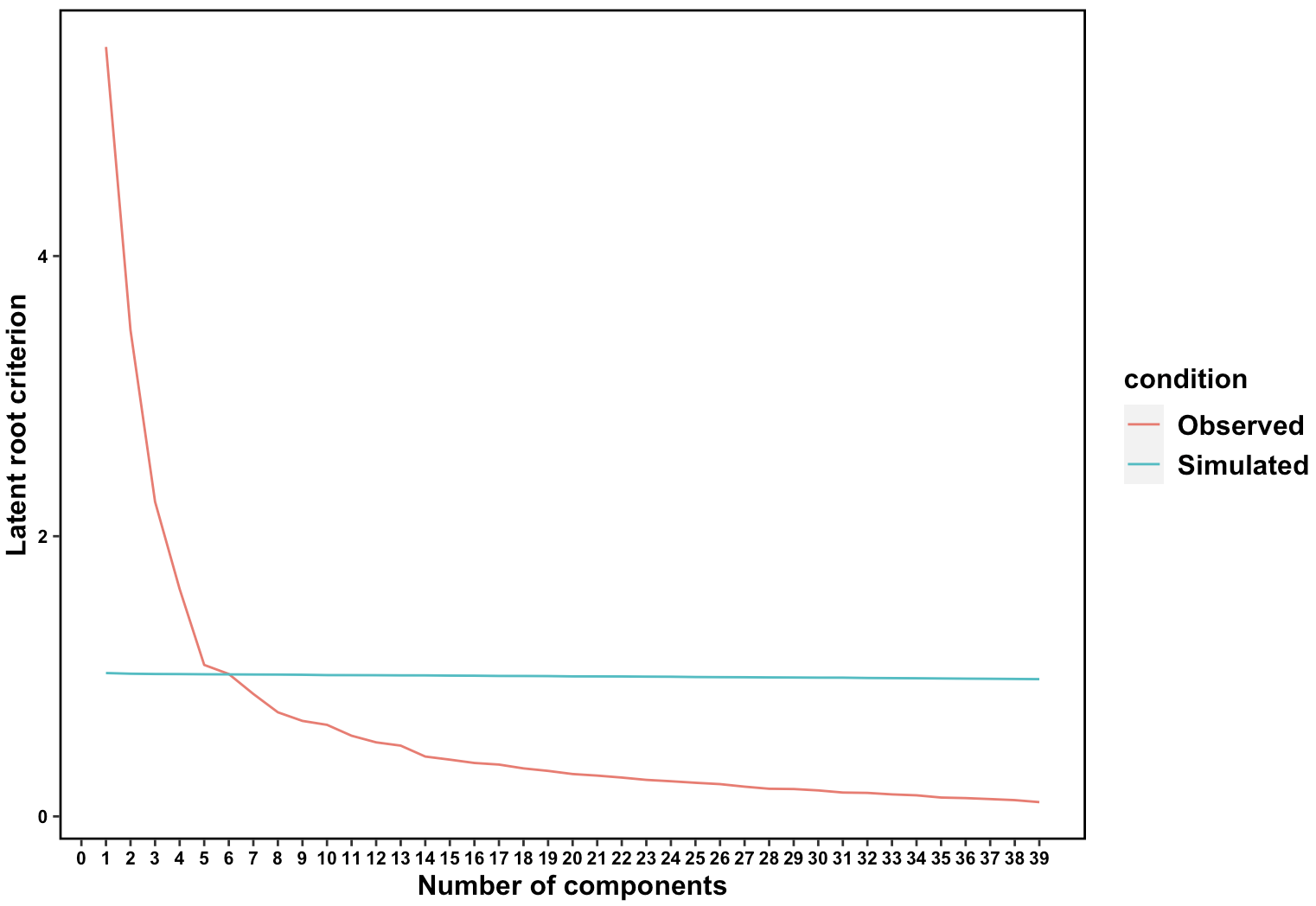
**

**Supplementary Figure 1. Visual depiction of the Horn’s analysis to determine the optimal number of EBs to retain for analysis.** The observed latent roots exceeded random (simulated) ones for the first five EBs. This means that they explained significantly more variance than expected by chance. EB = Eigenbrain.


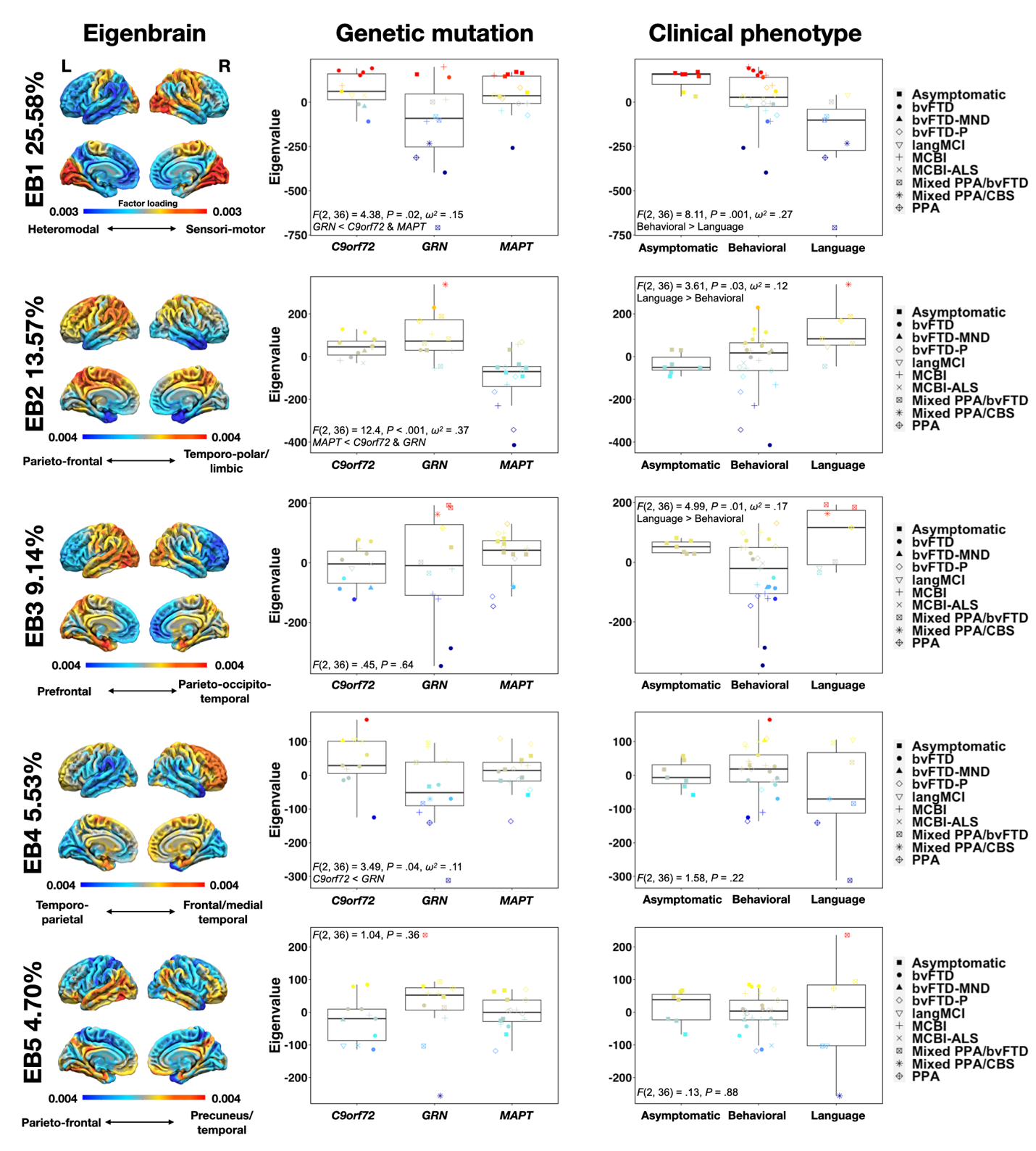


**Supplementary Figure 2. FDG-PET eigenbrains and group comparisons according to genetic mutation and predominant clinical phenotype.** The color bars represent positive (warm colors) and negative (cold colors) eigenvalues for each EB. These EBs reflect patterns of relative metabolism between two opposing poles of hyper- and hypometabolism. For each EBs, a negative eigenvalue is associated with lower metabolism in areas of cold colors relative to those with warm colors. EB = eigenbrain; bvFTD = behavioral variant frontotemporal dementia; MND = motor-neuron disease; P = parkinsonism; langMCI = language mild cognitive impairment; MCBI = mild cognitive and/or behavioral impairment; ALS = amyotrophic lateral sclerosis; PPA = primary progressive aphasia; CBS = corticobasal syndrome.

| Supplementary Table 1 Fine-grained characterization of symptomatic carriers | | | | | | |
| --- | --- | --- | --- | --- | --- | --- |
| Carrier | Mutation | Predominant phenotype | Additional features | Neurological examination | Speech pathology subspecialist evaluation | Neuropsychological evaluation |
| 1 | *C9orf72* | Behavioral | None | X |  |  |
| 2 | *C9orf72* | Behavioral | None | X |  | X |
| 3 | *C9orf72* | Behavioral | None | X |  |  |
| 4 | *C9orf72* | Behavioral | None | X |  |  |
| 5 | *C9orf72* | Behavioral | None | X |  | X |
| 6 | *C9orf72* | Behavioral | None | X |  |  |
| 7 | *C9orf72* | Behavioral | None | X |  |  |
| 8 | *C9orf72* | Behavioral | MND | X |  | X |
| 9 | *C9orf72* | Behavioral | None | X |  | X |
| 10 | *C9orf72* | Behavioral | MCBI + ALS | X |  |  |
| 11 | *C9orf72* | Language | Dysnomia of semantic type (MCI) | X |  | X |
| 12 | *GRN* | Behavioral | Mild agrammatism | X | X | X |
| 13 | *GRN* | Behavioral | None | X |  |  |
| 14 | *GRN* | Behavioral | None | X |  | X |
| 15 | *GRN* | Behavioral | None | X |  |  |
| 16 | *GRN* | Behavioral | None | X |  | X |
| 17 | *GRN* | Language | SD | X |  |  |
| 18 | *GRN* | Language | SD | X |  | X |
| 19 | *GRN* | Language | SD | X |  | X |
| 20 | *GRN* | Language | Aphasia + oral apraxia | X | X | X |
| 21 | *GRN* | Language | Logopenic aphasia + Corticobasal syndrome | X |  | X |
| 22 | *GRN* | Language | Non-fluent aphasia | X |  | X |
| 23 | *MAPT* | Behavioral | None | X |  |  |
| 24 | *MAPT* | Behavioral | Parkinsonism | X |  | X |
| 25 | *MAPT* | Behavioral | Parkinsonism | X |  |  |
| 26 | *MAPT* | Behavioral | Parkinsonism | X |  |  |
| 27 | *MAPT* | Behavioral | Parkinsonism | X |  |  |
| 28 | *MAPT* | Behavioral | Parkinsonism | X |  | X |
| 29 | *MAPT* | Behavioral | Parkinsonism | X |  | X |
| 30 | *MAPT* | Behavioral | MBCI | X |  |  |
| 31 | *MAPT* | Behavioral | MBCI | X |  |  |
| 32 | *MAPT* | Behavioral | MBCI | X |  |  |
| MCBI = Mild cognitive and behavioral impairment; MND = Motor-neuron disease; ALS = Amyotrophic lateral sclerosis; MCI = Mild cognitive impairment; SD = Semantic dementia. | | | | | | |
